# Prognostic impact of the combined effects of lipoprotein(a) and homocysteine in patients with premature myocardial infarction: a prospective cohort study

**DOI:** 10.3389/fnut.2026.1829711

**Published:** 2026-06-02

**Authors:** Yuhang Wang, Jingyu Liu, Xiaowei Li, Saidinisa Ruzemaimaiti, Changping Li, Yin Liu, Jing Gao

**Affiliations:** 1 Clinical School of Thoracic, Tianjin Medical University, Tianjin, China; 2 Department of Cardiology, Tianjin Chest Hospital, Tianjin, China; 3 School of Public Health, Tianjin Medical University, Tianjin, China; 4 Chest Hospital, Tianjin University, Tianjin, China; 5 Cardiovascular Institute, Tianjin Chest Hospital, Tianjin, China; 6 Tianjin Key Laboratory of Cardiovascular Emergency and Critical Care, Tianjin, China

**Keywords:** combined effects, homocysteine, lipoprotein(a), major adverse cardiovascular events, premature myocardial infarction

## Abstract

**Background:**

Lipoprotein(a) [Lp(a)] is a causal driver of atherosclerosis, yet the interaction between Lp(a)-driven lipid accumulation and a prothrombotic state induced by hyperhomocysteinemia has not been systematically investigated in this high-risk population. From a clinical nutrition perspective, homocysteine (HCY) is a critical modifiable metabolite influenced by B vitamins and folate, making this interaction particularly relevant for dietary or supplementation strategies. This study aims to systematically evaluate the independent and combined prognostic significance of Lp(a) and homocysteine (HCY) in premature myocardial infarction (PMI).

**Methods:**

This prospective cohort study enrolled 1741 PMI patients (aged ≤55 years) at Tianjin Chest Hospital (January 2018–June 2023). Restricted cubic spline (RCS) was used to explore nonlinearity between Lp(a)/HCY and major adverse cardiovascular events (MACE). HCY was analyzed both as a continuous variable and, for exploratory stratification, dichotomized at the clinically referenced threshold of 15 μmol/L. The cutoff for Lp(a) was pre-specified as 50 mg/dL based on clinical guidelines and further validated by maximally selected rank statistics (MSRS). Survival analyses assessed the effects of these factors on MACE. Net reclassification improvement (NRI) and integrated discrimination improvement (IDI) quantified incremental model value.

**Results:**

During a median follow-up of 19.6 months, 224 (12.87%) patients experienced MACE. RCS revealed a nonlinear association between Lp(a) and MACE (*p* = 0.003), whereas HCY showed no significant nonlinear trend. High Lp(a; HR = 2.266, 95% CI: 1.685–3.046, *p* < 0.001) and high HCY (HR = 1.597, 95% CI: 1.204–2.117, *p* = 0.001) are independent MACE risk factors. Compared with the low Lp(a) + low HCY group, the high Lp(a) + high HCY group had a 3.566-fold greater MACE risk (95% CI, 2.427–5.239, *p* < 0.001). Additive interaction analysis revealed a statistically significant interaction: RERI = 1.630 (95% CI, 0.314–2.945, *p* = 0.015), AP = 0.456 (95% CI, 0.197–0.716, *p* < 0.001), SI = 1.840 (95% CI, 0.960–2.719, *p* = 0.061).

**Conclusion:**

This study systematically characterizes the combined prognostic role of Lp(a) and HCY in PMI patients, validating a pragmatic “50–15” dual-threshold strategy for MACE risk stratification. The findings support a precision nutrition approach, suggesting that patients with dual elevation may benefit from targeted B-vitamin or folate supplementation as a low-cost adjunctive strategy.

## 1 Introduction

Premature myocardial infarction (PMI), typically defined as onset at ≤55 years of age, has garnered significant attention because of its early onset and substantial impact on family and social productivity (1, 2). Compared with elderly patients, PMI patients may exhibit distinct profiles of traditional atherosclerotic risk factors (e.g., hypertension, diabetes, and dyslipidemia) (3–5) and face a greater long-term recurrence risk (6), highlighting the urgency of identifying targeted risk factors and precise prevention strategies for this population.

Numerous epidemiological and genetic studies have confirmed that elevated Lp(a) is significantly associated with an increased risk of coronary artery disease (7), ischemic stroke (8), and aortic valve stenosis (9). In a large contemporary American cohort, elevated Lp(a) levels were independently associated with long-term major adverse cardiovascular events (MACE) in individuals with and without baseline atherosclerotic cardiovascular disease (ASCVD) (10). However, risk prediction based solely on Lp(a) remains insufficient in clinical practice, particularly in PMI patients. Some individuals with normal Lp(a) levels still experience adverse events.

Moreover, homocysteine (HCY), an intermediate product of methionine metabolism (11) whose levels are strongly influenced by dietary folate and B vitamins (B6, B12), has been investigated as a potential pathogenic factor for decades. Hyperhomocysteinemia (HHcy) accelerates atherosclerosis and thrombosis through multiple mechanisms, including increased platelet activation, promotion of oxidative stress (12), increased smooth muscle cell proliferation (13), and induction of endothelial dysfunction (14). Despite inconsistent results from large-scale interventional trials targeting HCY (e.g., folic acid supplementation) (15, 16), observational studies continue to support HCY as an independent risk factor, and emerging evidence suggests that the benefit of HCY reduction may be confined to high-risk subgroups with specific metabolic or genetic backgrounds (17).

Notably, Lp(a) and HCY share potential convergent pathogenic mechanisms. Theoretically, Lp(a)-mediated atherosclerotic plaque instability and HCY-induced endothelial injury with a procoagulant microenvironment may collectively accelerate disease progression. However, systematic prospective evidence regarding their combined role in PMI is lacking. Furthermore, from a nutritional standpoint, HCY is a sensitive functional indicator of folate and B-vitamin status, making it an attractive and modifiable target for dietary interventions. The potential for a cost-effective nutritional strategy to mitigate the excess risk associated with elevated Lp(a) warrants investigation.

Therefore, this prospective cohort study aimed to evaluate the independent prognostic value, combined effect, and interaction of Lp(a) and HCY in PMI patients. We propose a pragmatic “50–15” dual-threshold strategy based on clinically established cutoffs, with the goal of enabling precision nutritional and pharmacological risk stratification.

## 2 Methods

### 2.1 Study population

This prospective cohort study consecutively enrolled 2067 PMI patients who underwent coronary angiography (CAG) and were diagnosed at Tianjin Chest Hospital between January 2018 and June 2023. PMI included premature ST-elevation myocardial infarction (STEMI) and premature non-ST-segment elevation myocardial infarction (NSTEMI).

The inclusion criteria were as follows: (1) age ≤55 years; (2) first-time diagnosis of AMI; and (3) symptom onset ≤24 h. The diagnosis of AMI was based on the Fourth Universal Definition of Myocardial Infarction (18–20), requiring a serum cardiac biomarker (primarily troponin) elevation exceeding the 99th percentile of the reference limit, accompanied by at least one of the following clinical manifestations: (a) typical myocardial ischemia symptoms (persistent chest pain for 30 min unrelieved by 1–2 nitroglycerin tablets, associated with diaphoresis, nausea, vomiting, or pale complexion); (b) new-onset ischemic electrocardiographic (ECG) changes (including T-wave broadening, new ST-T segment changes, or left bundle branch block, or pathological Q waves on ECG); (c) imaging evidence of new regional myocardial dysfunction; and (d) coronary angiographic confirmation of coronary thrombosis.

The optimal cutoff value was determined based on clinical reference thresholds (14, 21–23). Lp(a) and HCY were dichotomized into low/high levels (Lp(a) ≤ 50 mg/dL, Lp(a) > 50 mg/dL, HCY ≤ 15 μmol/L, HCY > 15 μmol/L), and patients were stratified into four groups based on the combination of these biomarkers.

The exclusion criteria were as follows: (1) use of folic acid supplements (*n* = 32); (2) previous myocardial infarction (*n* = 36); (3) previous coronary revascularization (*n* = 47); (4) presence of any of the following: nonobstructive coronary myocardial infarction, congenital heart disease, moderate-to-severe valvular heart disease, aortic dissection, pulmonary embolism, severe liver or renal failure, severe inflammatory diseases, malignant tumors, thyroid diseases, or rheumatic diseases (*n* = 58); or (5) missing clinical data (*n* = 153).

The study protocol was approved by the Institutional Review Board of Tianjin Chest Hospital (No. 2017 KY-007-01) and conducted in accordance with the Declaration of Helsinki. All enrolled patients provided written informed consent prior to participation.

### 2.2 Data collection

Clinical data were extracted from the electronic medical record system upon the patient’s first admission, including age, sex, body mass index (BMI), smoking history, drinking history, admission heart rate (HR), admission systolic blood pressure (SBP) and diastolic blood pressure (DBP), past medical history (hypertension, diabetes, dyslipidemia, angina), family history of coronary artery disease (CAD), Killip classification, myocardial infarction (MI) type, echocardiographic parameters [left atrial diameter (LAD), left ventricular end-diastolic diameter (LVEDD), left ventricular ejection fraction (LVEF)], HCY, routine blood tests [C-reactive protein (CRP), white blood cells (WBC), neutrophils, lymphocytes, monocytes, red blood cells (RBC), hemoglobin (Hb), platelets (PLT), mean platelet volume (MPV), platelet distribution width (PDW), plateletcrit (PCT)], glycolipid metabolism indicators [total cholesterol (TC), triglycerides (TG), high-density lipoprotein (HDL), low-density lipoprotein (LDL), very-low-density lipoprotein (VLDL), apolipoprotein A1 (ApoA1), apolipoprotein B (ApoB), glycated hemoglobin (HbA1c), Lp(a)], renal function indicators [urea, creatinine (Cr), uric acid (UA)], liver function indicators [total bilirubin (TBil), direct bilirubin (DBil), alanine aminotransferase (ALT), aspartate aminotransferase (AST), alkaline phosphatase (ALP), gamma-glutamyl transferase (GGT), cholinesterase (CHE), lactate dehydrogenase (LDH), alpha-hydroxybutyrate dehydrogenase (*α*-HBDH)], cardiac injury markers [creatine kinase (CK), creatine kinase-MB (CK-MB), troponin T (TnT), B-type natriuretic peptide (BNP)], and coagulation indicators [D-dimer, fibrinogen (Fg)].

All laboratory results were obtained from fasting (12 h) peripheral blood samples collected during hospitalization. Plasma homocysteine (HCY) was measured via high-performance liquid chromatography with fluorescence detection. The mass concentration of lipoprotein(a) was determined via latex-enhanced turbidimetric immunoassay, which reports in mg/dL as per routine clinical practice in China.

### 2.3 Coronary angiography and treatment

Coronary angiography and, when necessary, percutaneous coronary intervention (PCI) were performed by at least two experienced interventional cardiologists. Coronary lesion characteristics, including the number of diseased vessels and the presence of occlusive lesions, were recorded. Multivessel disease (MVD) was defined as stenosis ≥ 40% in ≥ 2 major epicardial coronary arteries. In-hospital medications, including aspirin, clopidogrel/ticagrelor, statins, angiotensin-converting enzyme inhibitors (ACEI)/angiotensin II receptor blockers (ARB), and *β*-blockers, were also documented.

### 2.4 Study endpoint

The primary endpoint was MACE during follow-up, including cardiac death (*n* = 23), unplanned coronary revascularization (*n* = 83), heart failure readmission (*n* = 63), nonfatal recurrent MI (*n* = 30), and nonfatal stroke (*n* = 25). All patients were followed up by trained specialists via outpatient visits or telephone calls to record MACE occurrence post-MI. Cardiac death was defined as death due to sudden cardiac arrest, acute congestive heart failure, acute MI, severe arrhythmia, or other structural/functional heart diseases. Stroke was diagnosed on the basis of imaging findings or typical symptoms. Heart failure was defined according to the European Society of Cardiology guidelines as a clinical syndrome caused by structural or functional cardiac abnormalities leading to impaired ventricular filling and/or ejection, insufficient cardiac output to meet tissue metabolic demands, and manifestations such as dyspnea, exercise intolerance, and fluid retention. Recurrent AMI was diagnosed on the basis of chest pain, changes in myocardial enzyme levels, and ECG findings. Unplanned coronary revascularization was defined as revascularization driven by ischemic symptoms or pathological events, including unplanned PCI and coronary artery bypass grafting (CABG).

### 2.5 Statistical analysis

Statistical analyses were performed via SPSS 26.0 and R 4.3.1 software. Categorical variables are presented as n (%), and group differences were compared via the chi-square test or Fisher’s exact test. Continuous variables with a normal distribution and homogeneous variance are expressed as the means ± standard deviations and were compared via independent samples t tests. Nonnormally distributed or heteroscedastic continuous variables are presented as medians (interquartile ranges) and were compared via nonparametric rank-sum tests.

Restricted cubic spline (RCS) analysis was used to explore the nonlinear relationships between Lp(a)/HCY and MACE. For Lp(a), the optimal cut-off value was also explored using maximally selected rank statistics (MSRS), and sensitivity analyses compared the data-driven cut-off with the clinically defined 50 mg/dL threshold. For HCY, we analyzed HCY both as a continuous variable and dichotomized at the clinically referenced 15 μmol/L for exploratory subgroup stratification; the latter approach inevitably leads to information loss and is presented as a pragmatic clinical tool.

Kaplan–Meier curves with log-rank tests compared survival across subgroups. Multivariable Cox proportional hazards models were applied. Additive interaction was assessed by three metrics: relative excess risk due to interaction (RERI), attributable proportion (AP), and synergy index (SI). Net reclassification improvement (NRI) and integrated discrimination improvement (IDI) were calculated using 1,000 bootstrap samples.

Subgroup analyses were performed to evaluate the consistency of the combined Lp(a) and HCY effect across clinically relevant strata, including sex, type of myocardial infarction (STEMI vs. NSTEMI), hypertension status, diabetes status, and median-based cutoffs of white blood cell count, triglycerides, high-density lipoprotein, and D-dimer. For each subgroup, separate Cox models were fitted using the four-category combination variable (low Lp(a) + low HCY as reference) to estimate hazard ratios and 95% confidence intervals. Multiplicative interactions between Lp(a) and HCY were tested by including their product term in the model.

All tests were two-sided with *α* = 0.05.

## 3 Results

### 3.1 Baseline characteristics

A total of 1741 PMI patients who underwent coronary angiography were enrolled, including 1,533 males (88.05%) and 208 females (11.95%). During a median follow-up of 19.6 months (IQR: 12.7–35.2 months), 224 (12.87%) patients experienced MACE. Patients were stratified into four groups: Group 1 (low Lp(a) + low HCY, *n* = 995), Group 2 (low Lp(a) + high HCY, *n* = 489), Group 3 (high Lp(a) + low HCY, *n* = 148), and Group 4 (high Lp(a) + high HCY, *n* = 109; Table 1).

**Table 1:** Baseline characteristics of PMI patients stratified by Lp(a) and HCY levels.

Variables	Overall	Group 1 Low Lp(a) + Low HCY	Group 2 Low Lp(a) + High HCY	Group 3 High Lp(a) + Low HCY	Group 4 High Lp(a) + High HCY	*p* value
	*n* = 1741	*n* = 995	*n* = 489	*n* = 148	*n* = 109	
Age, years	42 (37, 44)	42 (38,44)	40 (36,44)	44 (39,48)	42 (38,45)	<0.001
Male, (%)	1,533 (88.05)	865 (86.93)	468 (95.71)	102 (68.92)	98 (89.90)	<0.001
BMI, kg/m^2^	25.88 (24.50, 27.78)	25.95 (24.62,27.76)	25.85 (24.40,27.78)	25.67 (24.70,27.24)	25.70 (24.61,27.78)	0.880
Smoking, (%)	1,079 (61.98)	575 (57.79)	347 (70.96)	78 (52.70)	79 (72.48)	<0.001
Drinking, (%)	566 (32.51)	303 (30.45)	185 (37.83)	33 (22.30)	45 (41.28)	<0.001
HR, bpm	75.00 (67.00, 86.00)	75.00 (67.00,85.00)	77.00 (68.00,88.00)	75.00 (66.00,84.00)	78.00 (69.00,85.00)	0.133
SBP, mmHg	131.00 (120.00, 144.00)	131.00 (120.00,144.00)	130.00 (120.00,144.00)	130.00 (118.00,140.00)	135.00 (120.00,152.00)	0.156
DBP, mmHg	80.00 (70.00, 90.00)	80.00 (71.50,90.00)	80.00 (70.00,91.00)	80.00 (70.00,90.00)	86.00 (72.00,96.00)	0.074
Past history, (%)						
Hypertension	768 (44.11)	429 (43.12)	201 (41.10)	76 (51.35)	62 (56.88)	0.006
Diabetes	549 (31.53)	361 (36.28)	109 (22.29)	57 (38.51)	22 (20.18)	<0.001
Hyperlipidemia	357 (20.51)	180 (18.09)	121 (24.74)	29 (19.59)	27 (24.77)	0.017
Angina	207 (11.89)	107 (10.75)	59 (12.07)	22 (14.86)	19 (17.43)	0.128
Family history of CAD	155 (8.90)	87 (8.74)	41 (8.38)	15 (10.14)	12 (11.01)	0.785
Killip classification, (%)						0.385
I	1,647 (94.60)	936 (94.07)	468 (95.71)	142 (95.95)	101 (92.66)	
≥II	94 (5.40)	59 (5.93)	21 (4.29)	6 (4.05)	8 (7.34)	
Type of MI, (%)						0.362
STEMI	1,254 (72.03)	720 (72.36)	351 (71.78)	107 (72.30)	76 (72.48)	
NSTEMI	487 (27.97)	275 (27.64)	138 (28.22)	41 (27.70)	33 (27.52)	
Laboratory data						
Cardiac ultrasound						
LAD, mm	36.00 (34.00, 39.00)	36.00 (34.00,39.00)	36.00 (34.00,39.00)	36.00 (33.75,39.00)	36.00 (34.00,39.00)	0.748
LVEDD, mm	51.00 (49.00, 55.00)	51.00 (48.00,54.50)	52.00 (49.00,55.00)	50.00 (48.00,54.25)	52.00 (49.00,55.00)	0.034
LVEF, %	52.00 (46.00, 57.00)	53.00 (47.00,57.00)	51.00 (46.00,56.00)	51.00 (45.00,58.00)	50.00 (44.00,55.00)	0.004
Blood routine						
CRP, mg/L	5.18 (2.37, 11.06)	4.92 (2.21,10.62)	5.41 (2.43,11.60)	5.22 (2.57,10.89)	6.46 (3.62,13.07)	0.017
WBC, 10^9/L	10.31 (8.48, 12.61)	10.12 (8.38,12.50)	10.80 (8.80,12.81)	9.88 (7.78,12.42)	10.50 (8.74,12.76)	0.004
Neutrophil, 10^9/L	7.58 (5.75, 9.90)	7.41 (5.67,9.75)	8.01 (6.29,10.26)	7.16 (5.45,9.46)	7.90 (6.13,10.00)	0.004
Lymphocyte, 10^9/L	1.84 (1.40, 2.35)	1.89 (1.44,2.40)	1.81 (1.36,2.30)	1.83 (1.44,2.25)	1.71 (1.28,2.20)	0.070
Monocyte, 10^9/L	0.60 (0.45, 0.77)	0.58 (0.43,0.77)	0.61 (0.48,0.80)	0.57 (0.43,0.78)	0.62 (0.46,0.78)	0.028
RBC, 10^12/L	4.86 (4.49, 5.18)	4.81 (4.45,5.17)	4.94 (4.64,5.21)	4.73 (4.33,5.00)	4.93 (4.57,5.27)	<0.001
Hb, g/L	147.00 (137.00, 157.00)	146.00 (136.00,156.00)	152.00 (142.00,160.00)	141.50 (131.00,152.00)	148.00 (141.00,158.00)	<0.001
PLT, 10^9/L	245.00 (209.00, 287.00)	244.00 (208.00,285.00)	241.00 (209.00,288.00)	251.00 (213.25,285.00)	250.00 (217.00,299.00)	0.600
MPV, fl	10.00 (9.40, 10.70)	10.00 (9.40,10.70)	9.90 (9.20,10.60)	10.00 (9.50,10.80)	9.90 (9.30,10.60)	0.052
PDW, %	12.20 (10.70, 14.90)	12.20 (10.70,14.50)	12.30 (10.80,15.90)	11.95 (10.80,14.22)	12.30 (10.70,15.10)	0.521
PCT, %	0.24 (0.21, 0.28)	0.25 (0.21,0.28)	0.24 (0.20,0.28)	0.25 (0.21,0.28)	0.25 (0.21,0.28)	0.180
Glycolipid metabolism indicators						
TC, mmol/L	4.83 (4.10, 5.55)	4.76 (4.07,5.46)	4.81 (4.09,5.53)	5.09 (4.36,5.88)	5.18 (4.37,5.98)	<0.001
TG, mmol/L	2.06 (1.46, 3.14)	2.11 (1.46,3.33)	2.06 (1.42,3.00)	1.98 (1.56,2.45)	2.01 (1.65,2.95)	0.113
HDL, mmol/L	0.93 (0.80, 1.08)	0.91 (0.79,1.06)	0.94 (0.81,1.08)	1.00 (0.85,1.17)	0.91 (0.76,1.09)	0.003
LDL, mmol/L	3.12 (2.43, 3.76)	2.99 (2.38,3.66)	3.14 (2.44,3.76)	3.46 (2.79,4.15)	3.60 (2.95,4.46)	<0.001
VLDL, mmol/L	0.58 (0.37, 0.88)	0.59 (0.38,0.91)	0.60 (0.37,0.88)	0.53 (0.35,0.78)	0.57 (0.40,0.83)	0.319
ApoA1, g/L	1.14 (1.01, 1.27)	1.14 (1.02,1.27)	1.13 (1.01,1.26)	1.14 (1.00,1.33)	1.09 (0.99,1.22)	0.105
ApoB, g/L	1.14 (0.94, 1.34)	1.13 (0.94,1.31)	1.12 (0.93,1.33)	1.17 (1.00,1.37)	1.22 (1.04,1.51)	<0.001
HbA1c, %	5.70 (5.40, 6.50)	5.80 (5.40,6.90)	5.60 (5.30,6.10)	5.80 (5.47,7.03)	5.60 (5.30,6.10)	<0.001
Kidney function indicators						
Urea, mmol/L	4.30 (3.50, 5.30)	4.30 (3.50,5.30)	4.30 (3.60,5.40)	4.20 (3.40,5.10)	4.90 (3.60,6.00)	0.015
Cr, umol/L	74.00 (64.00, 84.00)	71.00 (61.55,81.00)	78.00 (69.00,90.00)	71.22 (59.00,80.25)	82.00 (72.00,102.00)	<0.001
UA, umol/L	361.00 (297.00, 430.00)	355.00 (289.00,422.00)	368.00 (313.00,443.00)	343.50 (274.00,416.25)	411.00 (316.00,480.00)	<0.001
Liver function indicators						
TBil, umol/L	14.20 (10.20, 19.35)	13.90 (10.00,18.90)	15.00 (10.70,21.01)	13.50 (9.88,17.64)	14.80 (10.72,19.30)	0.006
DBil, umol/L	4.25 (2.60, 5.80)	4.10 (2.35,5.60)	4.50 (3.00,6.50)	4.00 (2.31,5.30)	4.29 (3.00,5.60)	<0.001
ALT, U/L	43.10 (28.00, 68.10)	41.20 (27.55,65.75)	48.70 (30.30,70.30)	40.65 (23.67,61.85)	41.90 (32.20,72.30)	0.003
AST, U/L	106.30 (44.90, 216.10)	101.30 (40.50,203.05)	121.80 (53.70,243.40)	103.90 (43.65,226.55)	115.50 (45.30,273.60)	0.002
ALP, U/L	75.00 (63.00, 91.00)	75.00 (62.00,90.00)	77.00 (66.00,93.00)	74.00 (62.75,88.25)	75.00 (65.00,90.00)	0.121
GGT, U/L	37.00 (24.90, 56.00)	35.50 (24.50,54.10)	40.00 (27.70,59.20)	30.85 (19.08,49.92)	38.00 (26.90,58.80)	<0.001
CHE, U/L	9228.00 (8164.00, 10403.00)	9223.00 (8184.50,10364.00)	9262.00 (8224.00,10445.00)	9107.00 (7882.00,10553.50)	9259.00 (8323.00,10231.00)	0.917
LDH, U/L	464.00 (267.00, 791.00)	421.00 (251.00,741.50)	491.00 (292.00,872.00)	538.00 (276.25,794.50)	564.00 (321.00,846.00)	<0.001
α-HBDH, U/L	419.00 (220.00, 790.00)	381.00 (205.50,725.50)	451.00 (241.00,880.00)	495.50 (227.75,804.75)	510.00 (293.00,830.00)	<0.001
Cardiac function indicators						
CK, U/L	1018.00 (347.00, 2171.00)	940.00 (310.50,1956.50)	1165.00 (410.00,2469.00)	1097.50 (363.50,2306.25)	1197.00 (428.00,2213.00)	0.011
CK-MB, U/L	87.00 (32.57, 184.00)	82.00 (30.00,174.50)	92.00 (36.32,197.00)	81.67 (30.75,193.99)	106.00 (35.00,195.52)	0.096
TnT, ug/L	2.67 (0.82, 7.59)	2.34 (0.66,6.25)	3.24 (1.08,9.55)	2.73 (0.86,7.93)	3.83 (1.25,10.00)	<0.001
BNP, pg./mL	152.69 (38.61, 440.99)	148.30 (36.47,392.20)	161.70 (37.09,493.30)	152.78 (44.48,357.19)	214.30 (60.06,817.10)	0.014
Coagulation indicators						
D-dimer, mg/L	0.28 (0.20, 0.41)	0.27 (0.19,0.40)	0.28 (0.20,0.40)	0.29 (0.22,0.45)	0.33 (0.24,0.56)	<0.001
Fg, g/L	3.32 (2.88, 3.91)	3.30 (2.87,3.91)	3.27 (2.84,3.81)	3.41 (3.02,4.09)	3.54 (3.05,4.19)	0.004
MVD, (%)	989 (56.81)	566 (56.88)	255 (52.15)	104 (70.27)	64 (58.72)	0.001
Coronary occlusion, (%)	1,007 (57.84)	560 (56.28)	289 (59.10)	98 (66.22)	60 (55.05)	0.116
Treatment, (%)						
Aspirin	1735 (99.66)	991 (99.60)	489 (100.00)	146 (98.65)	109 (100.00)	0.114
Clopidogrel/Ticagrelor	1736 (99.71)	990 (99.50)	489 (100.00)	148 (100.00)	109 (100.00)	0.409
Statins	1704 (97.87)	971 (97.59)	483 (98.77)	144 (97.30)	106 (97.25)	0.337
ACEI/ARB	1,169 (67.15)	670 (67.34)	331 (67.69)	92 (62.16)	76 (69.72)	0.557
β-Blocker	1,360 (78.12)	767 (77.09)	393 (80.37)	110 (74.32)	90 (82.57)	0.205
PCI status, (%)						0.001
No PCI performed	383 (22.00)	207 (20.80)	116 (23.72)	25 (16.89)	35 (32.11)	
Timely PCI	867 (49.80)	475 (47.74)	263 (53.78)	81 (54.73)	48 (44.04)	
Other PCI	491 (28.20)	313 (31.46)	110 (22.49)	42 (28.38)	26 (23.85)	
MACE, (%)	224 (12.87)	92 (9.25)	57 (11.66)	32 (21.62)	43 (39.45)	<0.001

Low Lp(a), Lp(a) ≤ 50 mg/dL; High Lp(a), Lp(a)>50 mg/dL; Low HCY, HCY≤15 μmol/L; High HCY, HCY>15 μmol/L. Lp(a), lipoprotein(a); HCY, homocysteine; BMI, body mass index; HR, heart rate; SBP, systolic blood pressure; DBP, diastolic blood pressure; CAD, coronary artery disease; MI, myocardial infarction; STEMI, ST-elevation myocardial infarction; NSTEMI, non-ST-elevation myocardial infarction; LAD, left atrium diameter; LVEDD, left ventricular end-diastolic diameter; LVEF: left ventricular ejection fraction; CRP, C-reactive protein; WBC, white blood cells; RBC, red blood cells; Hb, hemoglobin; PLT, platelet; MPV, mean platelet volume; PDW, platelet distribution width; PCT: plateletcrit; TC, total cholesterol; TG, triglycerides; HDL, high-density lipoprotein; LDL, low-density lipoprotein; VLDL, very-low-density lipoprotein; ApoA1, apolipoprotein A1; ApoB, apolipoprotein B; HbA1c, hemoglobin A1c; Cr, creatinine; UA, uric acid; TBil, total bilirubin; DBil, direct bilirubin; ALT, alanine aminotransferase; AST, aspartate aminotransferase; ALP, alkaline phosphatase; GGT, gamma-glutamyl transferase; CHE: cholinesterase; LDH, lactate dehydrogenase; α-HBDH, alpha-hydroxybutyrate dehydrogenase; CK, creatine kinase; CK-MB, creatine kinase MB; TnT, troponin T; BNP, B-type natriuretic peptide; Fg, fibrinogen; MVD, multiple vessel disease; ACEI, angiotensin-converting enzyme inhibitor; ARB, angiotensin receptor blocker; PCI, percutaneous coronary intervention; MACE, major adverse cardiovascular events.

Significant differences across the four groups were observed in age (*p* < 0.001), male sex (*p* < 0.001), smoking status (*p* < 0.001), drinking status (*p* < 0.001), hypertension status (*p* = 0.006), diabetes status (*p* < 0.001), and dyslipidemia status (*p* = 0.017). Group 4 had the highest proportions of smokers, drinkers, hypertensive patients, and dyslipidemic patients, whereas the proportion of diabetic patients was lowest in Group 4.

Significant intergroup differences were also noted in LVEDD (*p* = 0.034), LVEF (*p* = 0.004), CRP (*p* = 0.017), WBC (*p* = 0.004), neutrophils (*p* = 0.004), monocytes (*p* = 0.028), RBC (*p* < 0.001), Hb (*p* < 0.001), TC (*p* < 0.001), HDL (*p* = 0.003), LDL (*p* < 0.001), ApoB (*p* < 0.001), HbA1c (*p* < 0.001), urea (*p* = 0.015), Cr (*p* < 0.001), UA (*p* < 0.001), TBil (*p* = 0.006), DBil (*p* < 0.001), ALT (*p* = 0.003), AST (*p* = 0.002), GGT (*p* < 0.001), LDH (*p* < 0.001), *α*-HBDH (*p* < 0.001), CK (*p* = 0.011), TnT (*p* < 0.001), BNP (*p* = 0.014), D-dimer (*p* < 0.001), Fg (*p* = 0.004), MVD (*p* = 0.001), and PCI status (*p* = 0.001). Group 4 had the highest levels of LVEDD, monocytes, ApoB, urea, UA, and Fg. LVEF, CRP, TC, LDL, Cr, LDH, α-HBDH, CK, TnT, BNP, and D-dimer were lowest in Group 1 and highest in Group 4. Timely PCI was the most common intervention across all groups. The MACE incidence was lowest in Group 1 and highest in Group 4.

In the MACE group, the proportions of diabetic patients (*p* = 0.002) and MVD patients (*p* = 0.047) were significantly greater, the LAD was significantly greater (*p* = 0.036), and the LVEF was significantly lower (*p* = 0.001; Supplementary Table 1). Additionally, CRP (*p* = 0.006), ApoB (*p* = 0.041), urea (*p* = 0.011), DBil (*p* = 0.010), LDH (*p* = 0.028), *α*-HBDH (*p* = 0.048), TnT (*p* = 0.048), BNP (*p* = 0.008), D-dimer (*p* = 0.046), Lp(a; *p* < 0.001), and HCY (*p* < 0.001) were significantly elevated in the MACE group.

### 3.2 Association and stratification of Lp(a), HCY, and MACE risk

**Figure 1:**
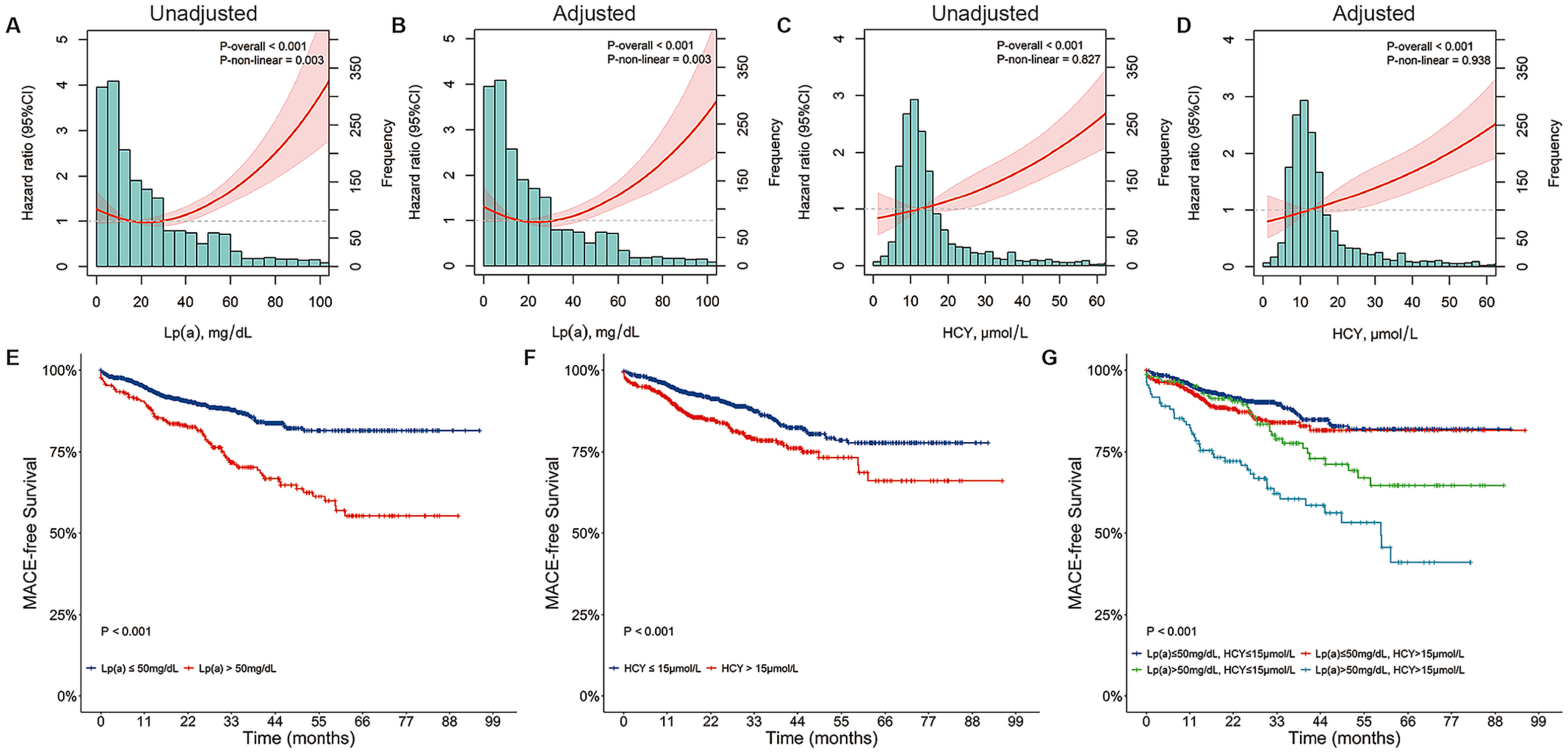
RCS and Kaplan–Meier curves for MACE risk associated with Lp(a) and HCY in PMI patients. **(A,B)** RCS curves for Lp(a) and MACE risk in the total population (unadjusted and adjusted); **(C,D)** RCS curves for HCY and MACE risk in the total population (unadjusted and adjusted). The red line represents the fit within the 95% confidence interval (shaded red area). Overall *p*-values and nonlinear *p*-values are provided. The green histograms show the frequency distributions and trends of Lp(a) and HCY. Adjusted for sex, age, body mass index, smoking status, drinking status, family history of coronary artery disease, Killip classification, type of myocardial infarction, left ventricular ejection fraction, C-reactive protein, low-density lipoprotein, creatine kinase, troponin T, B-type natriuretic peptide, D-dimer, fibrinogen, multivessel disease, and percutaneous coronary intervention status. **(E–G)** Kaplan–Meier curves with log-rank *p*-values demonstrating survival outcomes for Lp(a) and HCY, both independently and in combination, within the overall population. Lp(a), lipoprotein(a); HCY, homocysteine; MACE, major adverse cardiovascular events; CI, confidence interval.

Lp(a) and HCY exhibited nonnormal distributions (Shapiro–Wilk test, *p* < 0.05; Figures 1A–D). The RCS curves revealed significant associations between Lp(a)/HCY levels and MACE risk (P-overall<0.001; Figures 1A–D). Lp(a) showed a significant nonlinear correlation with MACE (P-nonlinear = 0.003; Figure 1B), with a “J-shaped” trend in the HR as Lp(a) levels increased. In the RCS curve, the cutoff values for Lp(a) and HCY are 47.99 mg/dL and 9.93 μmol/L, respectively. The MSRS identified an optimal Lp(a) cut-off value of 52.94 mg/dL, closely approximating the clinically predefined 50 mg/dL threshold (Supplementary Figure 1). In contrast, HCY was linearly associated with MACE (P-nonlinear = 0.938; Figure 1D).

Kaplan–Meier curves (Figures 1E–G) showed that patients with Lp(a) > 50 mg/dL or HCY > 15 μmol/L had significantly lower MACE-free survival (log-rank *p* < 0.001). Patients with concurrent high Lp(a) and high HCY had the worst prognosis.

### 3.3 Independent and interactive effects of Lp(a) and HCY on MACE risk in PMI patients

Multivariate Cox regression was used to systematically evaluate the impacts of Lp(a), HCY, and their combined stratification on MACE in PMI patients (Table 2). The proportional hazards (PH) assumption was statistically acceptable on the basis of residual analysis (*p* = 0.1199; Supplementary Figure 2).

**Table 2:** Multivariate Cox proportional hazards regression model for the independent and combined effects of Lp(a) and HCY on MACE risk in PMI patients.

Variables	Events/population (%)	Model 1		Model 2		Model 3	
		HR (95% CI)	*p* value	HR (95% CI)	*p* value	HR (95% CI)	*p* value
Continuous variables							
Lp(a), mg/dL		1.135 (1.087–1.184)	<0.001**	1.135 (1.087–1.185)	<0.001**	1.118 (1.070–1.169)	<0.001**
HCY, μmol/L		1.109 (1.081–1.138)	<0.001**	1.108 (1.080–1.137)	<0.001**	1.099 (1.070–1.128)	0.001**
Categorical variables							
Lp(a) ≤ 50 mg/dL	149/1484 (10.0)	Reference		Reference		Reference	
Lp(a)>50 mg/dL	75/257 (29.2)	2.358 (1.775–3.133)	<0.001**	2.373 (1.784–3.158)	<0.001**	2.266 (1.685–3.046)	<0.001**
HCY ≤ 15 μmol/L	124/1143 (10.8)	Reference		Reference		Reference	
HCY>15 μmol/L	100/598 (16.7)	1.778 (1.356–2.331)	<0.001**	1.818 (1.385–2.388)	<0.001**	1.597 (1.204–2.117)	0.001**
Individual combinations							
Low Lp(a), Low HCY	92/995 (9.2)	Reference		Reference		Reference	
Low Lp(a), High HCY	57/489 (11.7)	1.411 (1.010–1.970)	0.044*	1.445 (1.033–2.022)	0.031*	1.258 (0.890–1.778)	0.194
High Lp(a), Low HCY	32/148 (21.6)	1.763 (1.167–2.662)	0.007**	1.770 (1.170–2.679)	0.007**	1.682 (1.105–2.561)	0.015*
High Lp(a), High HCY	43/109 (39.4)	3.931 (2.733–5.655)	<0.001**	4.038 (2.799–5.826)	<0.001	3.566 (2.427–5.239)	<0.001**
Additive effects							
RERI		1.760 (0.367–3.153)	0.013*	1.825 (0.389–3.262)	0.013*	1.630 (0.314–2.945)	0.015*
AP		0.477 (0.195–0.700)	<0.001**	0.452 (0.201–0.702)	<0.001**	0.456 (0.197–0.716)	<0.001**
SI		1.810 (0.983–2.636)	0.055	1.824 (0.990–2.657)	0.053	1.840 (0.960–2.719)	0.061
Multiplicative effect		1.582 (0.897–2.789)	0.113	1.579 (0.893–2.792)	0.116	1.687 (0.951–2.991)	0.074

Model 1: Adjusted for sex, age; Model 2: Adjusted for Model 1 + body mass index, smoking, drinking, family history of coronary artery disease, killip classification, type of myocardial infarction; Model 3: Adjusted for Model 2 + left ventricular ejection fraction, C-reactive protein, low-density lipoprotein, creatinine, troponin T, B-type natriuretic peptide, D-dimer, fibrinogen, multiple vessel disease, percutaneous coronary intervention status. *p < 0.05, **p < 0.01. Low Lp(a) ≤ 50 mg/dL; high Lp(a) > 50 mg/dL; low HCY ≤15 μmol/L; high HCY >15 μmol/L. Lp(a), lipoprotein(a); HCY, homocysteine; HR, hazard ratio; CI, confidence interval; RERI, relative excess risk due to interaction; AP, attributable proportion due to interaction; SI, synergy index.

Elevated Lp(a; >50 mg/dL; HR = 2.266, 95% CI: 1.685–3.046; *p* < 0.001) and elevated HCY (>15 μmol/L; HR = 1.597, 95% CI: 1.204–2.117; *p* = 0.001) were independent risk factors for MACE in PMI patients (Table 2). We further examined the continuous relationship of Lp(a) and HCY with MACE. Both Lp(a; HR = 1.118, 95% CI: 1.070–1.169, *p* < 0.001) and HCY (HR = 1.099, 95% CI: 1.070–1.128, *p* = 0.001) were independently associated with MACE when modeled as continuous variables. Concurrent exposure to high Lp(a) and high HCY was associated with a significantly higher MACE risk (HR = 3.566, 95% CI: 2.427–5.239; *p*<0.001) compared to single-factor exposure.

The additive interaction metrics were as follows: RERI = 1.630 (95% CI, 0.314–2.945, *p* = 0.015), AP = 0.456 (95% CI, 0.197–0.716, *p* < 0.001), SI = 1.840 (95% CI, 0.960–2.719, *p* = 0.061). The RERI confidence interval does not cross zero and the *p*-value is <0.05, indicating a statistically significant additive interaction between Lp(a) and HCY. The AP shows that approximately 45.6% of the joint effect is attributable to the interaction. The SI point estimate (>1) supports synergy, although its confidence interval marginally includes 1 (*p* = 0.061; Table 2).

In sensitivity analyses, when we used the MSRS-derived optimal Lp(a) cutoff of 52.94 mg/dL instead of the clinical 50 mg/dL threshold, the results remained consistent. High Lp(a; >52.94 mg/dL) was independently associated with MACE (Model 3: HR = 2.513, 95% CI: 1.854–3.406, *p* < 0.001), and the dual-exposed group (high Lp(a) + high HCY) showed an even higher risk (HR = 4.080, 95% CI: 2.755–6.043, *p* < 0.001). Additive interaction metrics also indicated significant synergy (RERI = 1.855, 95% CI: 0.307–3.404, *p* = 0.019; AP = 0.455, 95% CI: 0.190–0.720, *p* < 0.001; Supplementary Table 2). These findings confirm the robustness of the “50–15” dual-threshold strategy.

The NRI and IDI were used to assess the incremental value of adding Lp(a) and/or HCY to the baseline clinical model (Model A; Supplementary Table 3). Models incorporating Lp(a) and/or HCY (Models B–E) showed varying degrees of improvement in NRI and IDI compared to Model A. Comparison of Model C (Model A + HCY) with Model B (Model A + Lp(a)) [NRI, −0.158 (95% CI −0.287 to −0.017); IDI, −0.025 (95% CI −0.038 to −0.012)] indicated a stronger contribution of Lp(a) to model performance. Model E (Model A + Lp(a) + HCY + Lp(a)HCY) demonstrated significant improvements over Models A–C. Although NRI [−0.072 (95% CI −0.199 to 0.071)] and IDI [−0.001 (95% CI −0.003 to 0.002)] did not differ significantly between Model E and Model D (Model A + Lp(a) + HCY), the C-index of Model E was slightly great (0.660 vs. 0.658), suggesting a minimal incremental value of the interaction term.

### 3.4 Subgroup analysis

**Figure 2:**
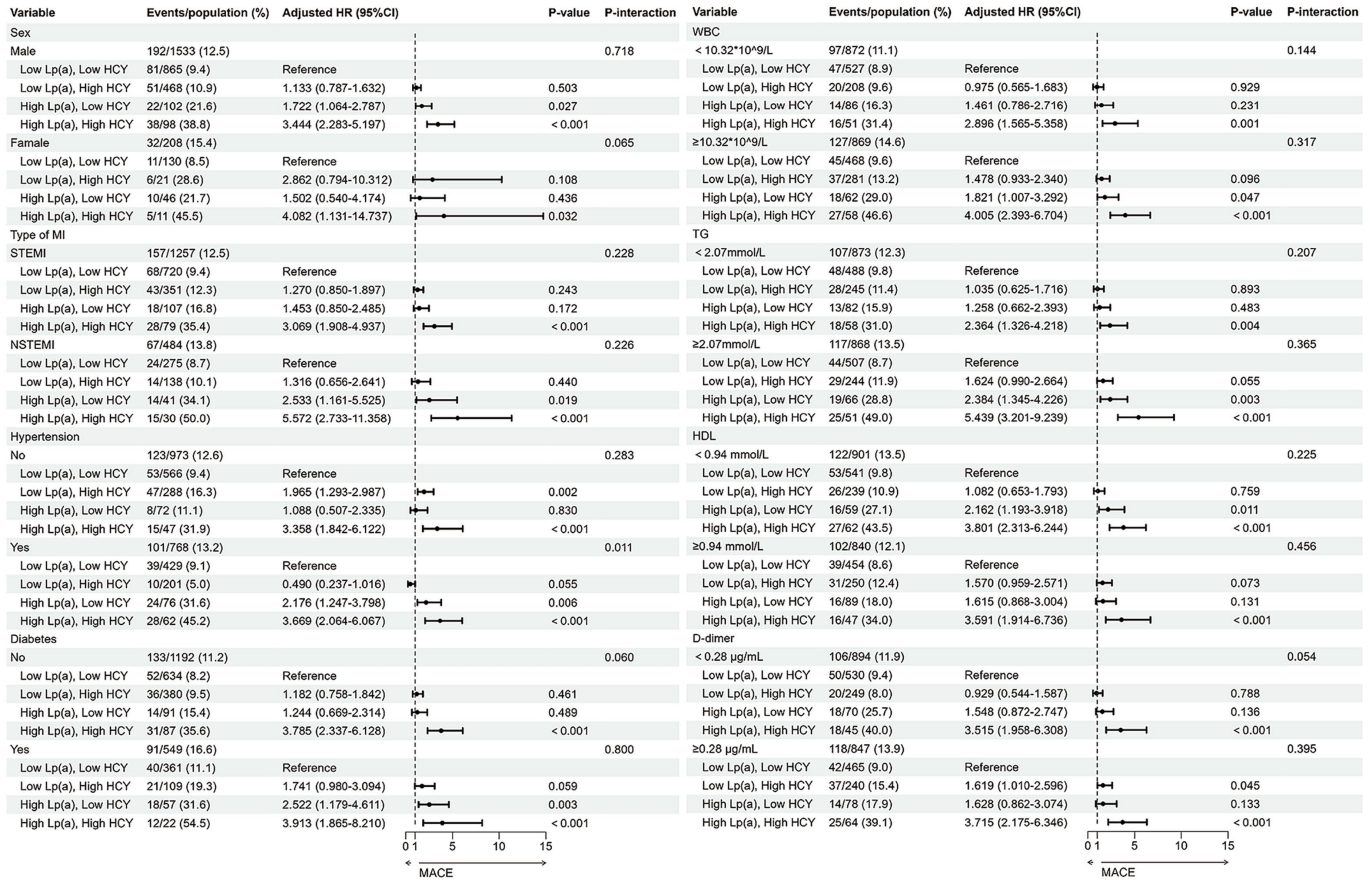
Subgroup analysis forest plot of the effect of combined Lp(a) and HCY on MACE in PMI patients. Adjusted for sex, age, body mass index, smoking, drinking, family history of coronary artery disease, Killip classification, type of myocardial infarction, left ventricular ejection fraction, C-reactive protein, low-density lipoprotein, creatine kinase, troponin T, B-type natriuretic peptide, D-dimer, fibrinogen, multivessel disease, and percutaneous coronary intervention status [Low Lp(a) ≤ 50 mg/dL; high Lp(a) > 50 mg/dL; low HCY ≤ 15 μmol/L; high HCY > 15 μmol/L]. HR, hazard ratio; CI, confidence interval; Lp(a), lipoprotein(a); HCY, homocysteine; STEMI, ST-elevation myocardial infarction; NSTEMI, non-ST-segment elevation myocardial infarction; WBC, white blood cell; TG, triglyceride; HDL, high-density lipoprotein.

Compared with males (HR = 3.444, 95% CI: 2.283–5.197, *p* < 0.001), females (HR = 4.082, 95% CI: 1.131–14.737, *p* = 0.032) exhibited a further increasing trend in MACE risk within the high Lp(a) + high HCY group (Figure 2). Within the NSTEMI subgroup (HR = 5.572, 95% CI: 2.733–11.358, *p* < 0.001), the MACE risk in the high Lp(a) + high HCY subgroup was greater than that in the STEMI subgroup (HR = 3.069, 95% CI: 1.908–4.937, *p* < 0.001). Regardless of hypertension or diabetes status, the high Lp(a) + high HCY subgroup had the highest MACE risk (no hypertension: HR = 3.358, 95% CI 1.842–6.122, *p* < 0.001; with hypertension: HR = 3.669, 95% CI: 2.064–6.067, *p* < 0.001; no diabetes: HR = 3.785, 95% CI 2.337–6.128, *p* < 0.001; with diabetes: HR = 3.913, 95% CI 1.865–8.210, *p* < 0.001), with a further upward trend in risk among patients with hypertension or diabetes. Additionally, significant multiplicative interactions between Lp(a) and HCY were observed in PMI patients with hypertension.

The WBC, TG, HDL and D-dimer levels were each stratified into two subgroups according to the median values used as cutoffs. The high Lp(a) + high HCY subgroup showed a further trend toward increased MACE risk in the high WBC, high TG, low HDL, and high D-dimer subgroups (Figure 2). In comparison, the highest hazard ratio was observed in the high Lp(a) + high HCY subgroup within the NSTEMI subgroup, followed by the high TG subgroup. The risk stratification value of combined Lp(a)-HCY grouping for MACE in PMI patients was consistently validated across all subgroups.

Component-specific analyses (Supplementary Table 4) revealed that the dual-exposed group had the strongest associations with unplanned coronary revascularization (Model 3: HR = 3.769, 95% CI: 2.024–7.018, *p* < 0.001) and heart failure readmission (HR = 2.498, 95% CI: 1.165–5.358, *p* = 0.019). For cardiac death, the dual-exposed group also showed a markedly increased risk (HR = 11.536, 95% CI: 2.970–44.813, *p* < 0.001), though with wide confidence intervals due to low event counts. For nonfatal recurrent MI, the dual-exposed group had a HR of 7.874 (95% CI: 2.863–21.656, *p* < 0.001). In contrast, no significant associations were observed for nonfatal stroke, possibly due to the small number of stroke events (*n* = 25) and insufficient statistical power. These component-specific findings suggest that the interaction effect of Lp(a) and HCY is most pronounced for ischemic-driven revascularization and heart failure, rather than equally across all MACE subtypes.

## 4 Discussion

This study is the first to systematically evaluate the independent and combined prognostic value of Lp(a) and HCY in PMI patients within a prospective cohort. The results demonstrated that elevated Lp(a; >50 mg/dL) and elevated HCY (>15 μmol/L) are independent risk factors for MACE, and that their concurrent elevation (high Lp(a) + high HCY subgroup) is associated with a markedly increased risk of MACE. Importantly, we observed a statistically significant additive interaction (RERI = 1.630, 95%CI 0.314–2.945, *p* = 0.015), indicating a interaction effect between Lp(a) and HCY on MACE risk. This synergy is further supported by the attributable proportion (AP = 0.456), suggesting that nearly half of the risk in the dual-exposed group can be attributed to the interaction. We propose and validate a pragmatic “50–15” dual-threshold combination, which holds potential clinical utility for precise risk stratification in PMI patients.

Lp(a) is a highly heritable biomarker. Despite significant ethnic variations in Lp(a) levels, elevated Lp(a) is independently associated with an increased risk of MI across all populations (24). Even in patients with ASCVD treated with statins, nonstatin agents, or combination therapies, Lp(a) has been implicated as a contributor to residual risk in those with low LDL levels (25). Lp(a) particles consist of an LDL-like core but are smaller and denser, facilitating infiltration into the vascular wall and extensive oxidation (26). Moreover, Lp(a) is a major carrier of proinflammatory oxidized phospholipids (OxPLs) and contains high levels of proinflammatory proteins (27).

Clinical risk cutoff values for Lp(a) vary across international and Chinese guidelines because of ethnic differences and research evidence. Most international guidelines define a high-risk cutoff of ≥50 mg/dL (125 nmol/L) (28, 29), with the National Lipid Association (NLA) Scientific Statement further classifying <30 mg/dL (75 nmol/L) as low risk and 30–50 mg/dL (75–125 nmol/L) as intermediate risk (30). In contrast, Chinese guidelines recommend a cutoff of ≥30 mg/dL (75 nmol/L) on the basis of the distribution of Lp(a) levels and evidence of risk in the Chinese population (31). Compared with the general population (32), the proportion of patients with elevated Lp(a) levels among those enrolled in this study was significantly greater. Therefore, the cutoff value for Lp(a) was defined as ≥50 mg/dL.

HCY is a key biomarker for cardiovascular diseases. A cross-sectional study revealed an association between HCY and cardiometabolic multimorbidity (CMM), which is defined as the coexistence of two or more of coronary heart diseases, stroke, and diabetes (33). Additionally, a study based on the NHANES database reported an association between HCY and subclinical myocardial injury in the general population, which further mediated cardiac death (34). A large community-based longitudinal study in Chinese residents revealed that elevated plasma HCY concentrations were associated with increased 10-year all-cause and cardiac mortality risks (35). The proportion of PMI patients with HCY > 15 μmol/L in this study (34.34%) was greater than that in the aforementioned community study (25.24%).

The pathogenic effects of Lp(a) are primarily mediated through pro-atherosclerosis (apolipoprotein(a) binds OxPL (27), promoting lipid deposition and vascular calcification (36)) and anti-fibrinolysis (apolipoprotein(a) competes with plasminogen for binding, inhibiting thrombolysis (37)). HCY induces vascular endothelial injury via oxidative stress-mediated NO depletion and endothelial apoptosis (38), activates monocytes–macrophages to release inflammatory factors (39), and promotes thrombosis by increasing platelet activation and increasing coagulation factor activity (40). The proinflammatory and plaque-promoting effects of Lp(a) depend on the “window” of endothelial injury caused by HCY to infiltrate the vascular wall; conversely, the prothrombotic state induced by HCY is further amplified by the antifibrinolytic effect of Lp(a), reflecting their interaction.

In female patients with PMI, the age of onset often approaches or exceeds the menopausal threshold, accompanied by a marked decrease in estrogen levels. Estrogen exerts vascular endothelial protective effects by inhibiting the release of inflammatory factors and reducing oxidative stress-induced damage (41). Additionally, it mitigates vascular injury, remodeling, and fibrosis following MI (41). Notably, Lp(a) levels are regulated by sex and hormonal status, with postmenopausal women potentially experiencing a further increases in plasma Lp(a) concentrations (42). Existing evidence has confirmed an association between elevated Lp(a) levels and the development of NSTEMI in younger populations (43). In contrast to the fully occlusive plaques observed during STEMI, the core pathological feature of NSTEMI is partially occlusive vulnerable coronary plaques, which are characterized by significant inflammatory cell infiltration and often present as incomplete thrombosis following plaque rupture or erosion (44). This pathological context reinforces the “inflammation-rupture” vicious cycle mediated by Lp(a) and HCY in vulnerable plaques. Thrombi in NSTEMI patients are predominantly platelet-rich “white thrombi,” and Lp(a) and HCY inhibit fibrinolytic activity and promote thrombosis, respectively, rendering patients more susceptible to adverse cardiovascular outcomes.

An elevated WBC count is a marker of an activated systemic inflammatory microenvironment. Lp(a) particles can continuously stimulate inflammatory cell activation, inducing the release of cytokines such as IL-1 (45) and exacerbating intraplaque inflammatory infiltration. HCY further aggravates endothelial injury and inflammatory factor release, forming a positive feedback loop of “inflammation–endothelial injury–Lp(a) deposition”. Elevated TG is often accompanied by increased triglyceride-rich lipoproteins (TRLs) (46); dyslipidemia provides a “substrate foundation” for the plaque-promoting effect of Lp(a), with TRLs and Lp(a) codepositing to promote lipid core formation (25). HCY amplifies this deposition effect through endothelial injury, ultimately accelerating the formation and rupture of vulnerable plaques. HDL can inhibit OxPL oxidation of Lp(a) via apoA1 (47), reducing its proinflammatory activity, and promoting the phosphorylation of endothelial nitric oxide synthase (eNOS) to release NO precursors and repair endothelial tight junctions (48). In the low HDL subgroup, this protective effect is significantly attenuated, leading to enhanced pro-inflammatory and plaquepromoting effects, as well as ineffective repair of endothelial injury. Consequently, the combined effect of Lp(a) and HCY is amplified due to the loss of HDL-mediated “buffering.” D-dimer is a degradation product of cross-linked fibrin; its elevation not only reflects acute thrombotic events but also indicates a chronic prothrombotic state characterized by a coagulation–fibrinolysis imbalance (49). Lp(a) upregulates the expression of plasminogen activator inhibitor-1 (PAI-1), inhibiting the activity of tissue-type plasminogen activator (t-PA) (50). At this stage, elevated HCY can induce significant changes in fibrin structure, promoting the formation of dense, rigid thrombi with reduced permeability and impaired fibrinolytic sensitivity (51), thereby fostering a prothrombotic environment. This phenomenon forms a “dual drive” with the antifibrinolytic effect of Lp(a), facilitating the progression of a prothrombotic state to clinical thrombotic events.

Several drugs targeting Lp(a) reduction, including antisense oligonucleotides (ASOs), small interfering RNAs (siRNAs), and small molecules, are currently in clinical trials (52). ASOs are short single-stranded synthetic RNA or DNA molecules that regulate genes and their variants by binding to complementary sequences, inhibiting apolipoprotein(a) production and ultimately reducing Lp(a) levels (53). Specific siRNAs reduce plasma Lp(a) levels by interfering with Lp(a) synthesis in the liver (52). Muvalaplin is the first oral small-molecule drug that reduces Lp(a) levels by targeting Lp(a) particle assembly rather than apolipoprotein(a) expression (54). Current drugs for reducing HCY mainly include B vitamins and trimethylglycine, which promote HCY metabolism through methylation (55). However, guidelines only recommend intervention for patients with H-type hypertension and high stroke risk (56), with no clear indications for the treatment of isolated hyperhomocysteinemia. Clinicians often do not actively recommend treatment due to insufficient evidence of benefit.

The component-specific analyses provide further insight. The dual-exposed group showed the highest risk for unplanned revascularization and heart failure readmission, which aligns with the known roles of Lp(a) in promoting plaque progression and HCY in impairing endothelial repair. Conversely, the lack of a significant association with nonfatal stroke may reflect the smaller number of stroke events or the possibility that the Lp(a)-HCY interaction is more relevant to coronary and heart failure outcomes than to cerebrovascular events. Future studies with larger sample sizes and longer follow-up are needed to clarify these subtype-specific effects.

This study has several limitations. First, as a single-center prospective cohort study, future multicenter, large-sample studies are needed to validate the generalizability of combined Lp(a) and HCY testing and further refine the combined risk assessment model for PMI subgroups with different comorbidities (e.g., chronic kidney disease). Second, biomarkers were only measured at baseline without repeated assessments. Although HCY and Lp(a) have been shown to be relatively stable due to genetic factors in previous studies, long-term follow-up is needed to explore the impact of combined Lp(a) and HCY exposure trajectories on MACE risk in PMI patients. Third, this study did not explore the impact of genetic factors (e.g., KIV-2 repeat sequences in the Lp(a) and MTHFR/CBS genes in HCY metabolism) on HCY and Lp(a) levels and their interactions. Future studies integrating genomics and lipid metabolomics are needed to elucidate the molecular mechanisms underlying their interaction. Fourth, data on dietary patterns, socioeconomic status, and specific serum levels of vitamin B12 and folate were not systematically collected. Fifth, the use of different units for measuring Lp(a; mg/dL and nmol/L) may limit the ability to compare results across studies. Sixth, since this study is purely observational, the PMI itself may have a slight effect on HCY and Lp(a) levels, which could introduce variability.

## 5 Conclusion

This study is the first to reveal the combined role of Lp(a) and HCY in adverse outcomes of PMI patients, validating a “50–15” dual threshold that significantly optimizes MACE risk stratification. PMI patients with high Lp(a) + high HCY levels have the poorest survival prognosis. A significant additive interaction exists between Lp(a) and HCY. The “50–15” dual threshold provides a novel target for precise risk intervention and a new strategy for the clinical management of PMI, with implications for precision nutrition.

## Data Availability

The data analyzed in this study is subject to the following licenses/restrictions: the datasets generated and/or analyzed during the current study are available from the corresponding author upon reasonable request. Requests to access these datasets should be directed to Jing Gao, gaojing2089@163.com.
