# Supplementary material for: Prognostic impact of the combined effects of lipoprotein(a) and homocysteine in patients with premature myocardial infarction: a prospective cohort study
Source: Front Nutr. 2026 Jun 2;13:1829711. doi: 10.3389/fnut.2026.1829711 (PMC13268957; doi:10.3389/fnut.2026.1829711)
Supplement: Supplementary file 1 [file Table_1.docx]

Supplementary Material

**Supplementary Table 1.** Baseline characteristics of PMI patients stratified by non-MACE and MACE status.

| **Variables** | **Overall** | **non-MACE** | **MACE** | **P value** |
| --- | --- | --- | --- | --- |
|  | **n=1741** | **n=1517** | **n=224** |  |
| Age, years | 42 (37, 44) | 42 (37, 44) | 42 (38, 45) | 0.121 |
| Male, (%) | 1533 (88.05) | 1341 (88.40) | 192 (85.71) | 0.248 |
| BMI, kg/m² | 25.88 (24.50, 27.78) | 25.95 (24.50, 27.72) | 25.58 (24.39, 28.40) | 0.915 |
| Smoking, (%) | 1079 (61.98) | 934 (61.57) | 145 (64.73) | 0.363 |
| Drinking, (%) | 566 (32.51) | 493 (32.50) | 73 (32.59) | 0.978 |
| HR, bpm | 75.00 (67.00, 86.00) | 75.00 (67.00, 85.00) | 76.00 (69.00, 86.00) | 0.138 |
| SBP, mmHg | 131.00 (120.00, 144.00) | 131.00 (120.00, 144.00) | 130.00 (119.00, 145.00) | 0.473 |
| DBP, mmHg | 80.00 (70.00, 90.00) | 80.00 (71.00, 90.00) | 80.00 (70.00, 90.00) | 0.490 |
| **Past history, (%)** |  |  |  |  |
| Hypertension | 768 (44.11) | 667 (43.97) | 101 (45.09) | 0.752 |
| Diabetes | 549 (31.53) | 458 (30.19) | 91 (40.62) | 0.002** |
| Hyperlipidemia | 357 (20.51) | 319 (21.03) | 38 (16.96) | 0.160 |
| Angina | 207 (11.89) | 176 (11.60) | 31 (13.84) | 0.334 |
| Family history of CAD | 155 (8.90) | 133 (8.77) | 22 (9.82) | 0.605 |
| **Killip classification, (%)** |  |  |  | 0.120 |
| I | 1647 (94.60) | 1440 (94.92) | 207 (92.41) |  |
| ≥II | 94 (5.40) | 77 (5.08) | 17 (7.59) |  |
| **Type of MI, (%)** |  |  |  | 0.489 |
| STEMI | 1254 (72.03) | 1097 (72.31) | 157 (70.09) |  |
| NSTEMI | 487 (27.97) | 420 (27.69) | 67 (29.91) |  |
| **Laboratory data** |  |  |  |  |
| **Cardiac ultrasound** |  |  |  |  |
| LAD, mm | 36.00 (34.00, 39.00) | 36.00 (34.00, 39.00) | 37.00 (34.00, 39.00) | 0.036* |
| LVEDD, mm | 51.00 (49.00, 55.00) | 51.00 (49.00, 55.00) | 51.50 (48.00, 56.00) | 0.335 |
| LVEF, % | 52.00 (46.00, 57.00) | 52.00 (47.00, 57.00) | 50.00 (42.00, 56.00) | 0.001** |
| **Blood routine** |  |  |  |  |
| CRP, mg/L | 5.18 (2.37, 11.06) | 5.08 (2.22, 11.02) | 5.99 (3.10, 12.70) | 0.006** |
| WBC, 10^9/L | 10.31 (8.48, 12.61) | 10.24 (8.45, 12.58) | 10.68 (8.74, 12.85) | 0.063 |
| Neutrophil, 10^9/L | 7.58 (5.75, 9.90) | 7.53 (5.71, 9.86) | 8.07 (6.04, 9.92) | 0.060 |
| Lymphocyte, 10^9/L | 1.84 (1.40, 2.35) | 1.84 (1.40, 2.34) | 1.84 (1.38, 2.37) | 0.804 |
| Monocyte, 10^9/L | 0.60 (0.45, 0.77) | 0.60 (0.45, 0.77) | 0.63 (0.45, 0.80) | 0.204 |
| RBC, 10^12/L | 4.86 (4.49, 5.18) | 4.85 (4.49, 5.17) | 4.89 (4.46, 5.18) | 0.853 |
| Hb, g/L | 147.00 (137.00, 157.00) | 147.00 (138.00, 157.00) | 147.00 (136.00, 157.00) | 0.603 |
| PLT, 10^9/L | 245.00 (209.00, 287.00) | 244.00 (209.00, 286.00) | 247.00 (208.75, 288.50) | 0.626 |
| MPV, fl | 10.00 (9.40, 10.70) | 10.00 (9.30, 10.70) | 10.00 (9.50, 10.70) | 0.383 |
| PDW, % | 12.20 (10.70, 14.90) | 12.20 (10.70, 14.90) | 12.25 (10.80, 14.48) | 0.832 |
| PCT, % | 0.24 (0.21, 0.28) | 0.24 (0.21, 0.28) | 0.25 (0.21, 0.29) | 0.158 |
| **Glycolipid metabolism indicators** |  |  |  |  |
| TC, mmol/L | 4.83 (4.10, 5.55) | 4.81 (4.09, 5.52) | 4.97 (4.18, 5.70) | 0.086 |
| TG, mmol/L | 2.06 (1.46, 3.14) | 2.05 (1.46, 3.15) | 2.11 (1.52, 3.09) | 0.512 |
| HDL, mmol/L | 0.93 (0.80, 1.08) | 0.93 (0.80, 1.08) | 0.92 (0.78, 1.08) | 0.251 |
| LDL, mmol/L | 3.12 (2.43, 3.76) | 3.10 (2.43, 3.73) | 3.19 (2.46, 3.99) | 0.067 |
| VLDL, mmol/L | 0.58 (0.37, 0.88) | 0.58 (0.37, 0.88) | 0.59 (0.39, 0.86) | 0.858 |
| ApoA1, g/L | 1.14 (1.01, 1.27) | 1.14 (1.01, 1.27) | 1.12 (1.00, 1.27) | 0.311 |
| ApoB, g/L | 1.14 (0.94, 1.34) | 1.13 (0.94, 1.33) | 1.17 (1.01, 1.39) | 0.041* |
| HbA1c, % | 5.70 (5.40, 6.50) | 5.70 (5.40, 6.40) | 5.70 (5.30, 7.00) | 0.886 |
| **Kidney function indicators** |  |  |  |  |
| Urea, mmol/L | 4.30 (3.50, 5.30) | 4.30 (3.50, 5.30) | 4.60 (3.70, 5.70) | 0.011* |
| Cr, μmol/L | 74.00 (64.00, 84.00) | 74.00 (64.00, 84.00) | 76.00 (64.00, 89.00) | 0.108 |
| UA, μmol/L | 361.00 (297.00, 430.00) | 360.00 (297.00, 429.00) | 363.00 (294.50, 445.25) | 0.431 |
| **Liver function indicators** |  |  |  |  |
| TBil, μmol/L | 14.20 (10.20, 19.35) | 14.10 (10.19, 19.30) | 14.60 (10.50, 19.70) | 0.468 |
| DBil, μmol/L | 4.25 (2.60, 5.80) | 4.20 (2.50, 5.80) | 4.60 (3.30, 6.23) | 0.010* |
| ALT, U/L | 43.10 (28.00, 68.10) | 43.10 (27.50, 67.90) | 43.45 (31.30, 73.80) | 0.233 |
| AST, U/L | 106.30 (44.90, 216.10) | 105.00 (43.50, 215.50) | 119.00 (52.90, 223.55) | 0.098 |
| ALP, U/L | 75.00 (63.00, 91.00) | 76.00 (63.00, 91.00) | 74.00 (63.00, 88.00) | 0.148 |
| GGT, U/L | 37.00 (24.90, 56.00) | 36.40 (24.70, 55.90) | 40.00 (25.67, 58.02) | 0.246 |
| CHE, U/L | 9228.00 (8164.00, 10403.00) | 9224.00 (8145.00, 10381.00) | 9257.50 (8252.00, 10486.50) | 0.553 |
| LDH, U/L | 464.00 (267.00, 791.00) | 458.00 (261.00, 780.00) | 488.50 (317.00, 834.75) | 0.028* |
| α-HBDH, U/L | 419.00 (220.00, 790.00) | 413.00 (215.00, 784.00) | 451.00 (274.75, 826.25) | 0.048* |
| **Cardiac Function Indicators** |  |  |  |  |
| CK, U/L | 1018.00 (347.00, 2171.00) | 991.00 (340.00, 2200.00) | 1190.00 (416.50, 2073.50) | 0.385 |
| CK-MB, U/L | 87.00 (32.57, 184.00) | 85.00 (32.00, 184.00) | 99.00 (36.75, 187.75) | 0.438 |
| TnT, μg/L | 2.67 (0.82, 7.59) | 2.61 (0.78, 7.38) | 3.20 (1.19, 8.72) | 0.048* |
| BNP, pg/mL | 152.69 (38.61, 440.99) | 150.90 (38.85, 402.00) | 200.88 (34.73, 741.35) | 0.008** |
| **Coagulation indicators** |  |  |  |  |
| D-dimer, μg/mL | 0.28 (0.20, 0.41) | 0.28 (0.20, 0.40) | 0.29 (0.21, 0.51) | 0.046* |
| Fg, g/L | 3.32 (2.88, 3.91) | 3.31 (2.86, 3.90) | 3.42 (2.95, 3.92) | 0.050 |
| **MVD, (%)** | 989 (56.81) | 848 (55.90) | 141 (62.95) | 0.047* |
| **Coronary occlusion, (%)** | 1007 (57.84) | 884 (58.27) | 123 (54.91) | 0.341 |
| **Treatment, (%)** |  |  |  |  |
| Aspirin | 1735 (99.66) | 1512 (99.67) | 223 (99.55) | 0.563 |
| Clopidogrel/Ticagrelor | 1736 (99.71) | 1512 (99.67) | 224 (100.00) | 1.000 |
| Statins | 1704 (97.87) | 1486 (97.96) | 218 (97.32) | 0.714 |
| ACEI/ARB | 1169 (67.15) | 1014 (66.84) | 155 (69.20) | 0.484 |
| β-Blocker | 1360 (78.12) | 1181 (77.85) | 179 (79.91) | 0.486 |
| **PCI status, (%)** |  |  |  | 0.101 |
| No PCI performed | 383 (22.00) | 325 (21.42) | 58 (25.89) |  |
| Timely PCI | 867 (49.80) | 752 (49.57) | 115 (51.34) |  |
| Other PCI | 491 (28.20) | 440 (29.00) | 51 (22.77) |  |
| **Lp(a), mg/dL** | 15.77 (6.63, 33.75) | 15.03 (6.50, 31.25) | 21.15 (7.38, 58.19) | <0.001** |
| **HCY, μmol/L** | 12.50 (9.52, 18.20) | 12.37 (9.36, 17.80) | 14.50 (10.52, 20.08) | <0.001** |
| Footnotes: *p < 0.05, **p < 0.01. BMI, body mass index; HR, heart rate; SBP, systolic blood pressure; DBP, diastolic blood pressure; CAD, coronary artery disease; MI, myocardial infarction; STEMI, ST-elevation myocardial infarction; NSTEMI, non-ST-elevation myocardial infarction; LAD, left atrium diameter; LVEDD, left ventricular end-diastolic diameter; LVEF: left ventricular ejection fraction; CRP, C-reactive protein; WBC, white blood cells; RBC, red blood cells; Hb, hemoglobin; PLT, platelet; MPV, mean platelet volume; PDW, platelet distribution width; PCT: plateletcrit; TC, total cholesterol; TG, triglycerides; HDL, high-density lipoprotein; LDL, low-density lipoprotein; VLDL, very-low-density lipoprotein; ApoA1, apolipoprotein A1; ApoB, apolipoprotein B; HbA1c, hemoglobin A1c; Cr, creatinine; UA, uric acid; TBil, total bilirubin; DBil, direct bilirubin; ALT, alanine aminotransferase; AST, aspartate aminotransferase; ALP, alkaline phosphatase; GGT, gamma-glutamyl transferase; CHE: cholinesterase; LDH, lactate dehydrogenase; α-HBDH, alpha-hydroxybutyrate dehydrogenase; CK, creatine kinase; CK-MB, creatine kinase MB; TnT, troponin T; BNP, B-type natriuretic peptide; Fg, fibrinogen; MVD, multiple vessel disease; ACEI, angiotensin-converting enzyme inhibitor; ARB, angiotensin receptor blocker; PCI, percutaneous coronary intervention; MACE, major adverse cardiovascular events; Lp(a), lipoprotein(a); HCY, homocysteine. | | | | |

**Supplementary Table 2. Sensitivity analysis using MSRS-derived Lp(a) cutoff of 52.94 mg/dL.**

|  |  | **Model 1** | | **Model 2** | | **Model 3** | |
| --- | --- | --- | --- | --- | --- | --- | --- |
| **Group** | **Events/population (%)** | **HR (95% CI)** | **P value** | **HR (95% CI)** | **P value** | **HR (95% CI)** | **P value** |
| Lp(a)≤52.94mg/dL | 152/1522 (10.0) | Reference | | Reference | | Reference | |
| Lp(a)＞52.94mg/dL | 72/219 (32.9) | 2.564 (1.919–3.425) | ＜0.001** | 2.626 (1.963–3.513) | ＜0.001** | 2.513 (1.854–3.406) | ＜0.001** |
| HCY≤15μmol/L | 124/1143 (10.8) | Reference | | Reference | | Reference | |
| HCY＞15μmol/L | 100/598 (16.7) | 1.778 (1.356–2.331) | ＜0.001** | 1.818 (1.385–2.388) | ＜0.001** | 1.597 (1.204–2.117) | 0.001** |
| **Individual combinations** |  |  |  |  |  |  |  |
| Low Lp(a), Low HCY | 94/1020 (9.2) | Reference | | Reference | | Reference | |
| Low Lp(a), High HCY | 58/502 (11.6) | 1.399 (1.005–1.948) | 0.047* | 1.440 (1.033–2.008) | 0.031* | 1.266 (0.899–1.783) | 0.177 |
| High Lp(a), Low HCY | 30/123 (24.4) | 2.032 (1.331–3.101) | 0.001** | 2.096 (1.369–3.209) | ＜0.001** | 1.958 (1.270–3.019) | 0.002** |
| High Lp(a), High HCY | 42/96 (43.8) | 4.486 (3.112–6.466) | ＜0.001** | 4.687 (3.237–6.786) | ＜0.001** | 4.080 (2.755–6.043) | ＜0.001** |
| **Additive effects** |  |  |  |  |  |  |  |
| **RERI** |  | 2.055 (0.441–3.669) | 0.013* | 2.151 (0.450–3.852) | 0.013* | 1.855 (0.307–3.404) | 0.019* |
| **AP** |  | 0.458 (0.206–0.710) | ＜0.001** | 0.459 (0.207–0.711) | ＜0.001** | 0.455 (0.190–0.720) | ＜0.001** |
| **SI** |  | 1.845 (0.989–2.702) | 0.053 | 1.848 (0.988–2.708) | 0.053 | 1.834 (0.943–2.725) | 0.067 |
| **Multiplicative effect** |  | 1.578 (0.887–2.807) | 0.121 | 1.553 (0.869–2.774) | 0.137 | 1.645 (0.917–2.951) | 0.095 |
| Model 1: Adjusted for sex, age; Model 2: Adjusted for Model 1 + body mass index, smoking, drinking, family history of coronary artery disease, killip classification, type of myocardial infarction; Model 3: Adjusted for Model 2 + left ventricular ejection fraction, C-reactive protein, low-density lipoprotein, creatinine, troponin T, B-type natriuretic peptide, D-dimer, fibrinogen, multiple vessel disease, percutaneous coronary intervention status. Foototes: *P<0.05, **P<0.01.Low Lp(a) ≤52.94 mg/dL; high Lp(a) >52.94 mg/dL; low HCY ≤15 μmol/L; high HCY >15 μmol/L. Lp(a), lipoprotein(a); HCY, homocysteine; HR, hazard ratio; CI, confidence interval; RERI, relative excess risk due to interaction; AP, attributable proportion due to interaction; SI, synergy index. | | | | | | | |

| **Supplementary Table 3.** Incremental values of Lp(a) and HCY as covariates added to the baseline clinical model (Model A).   \|  \|  \| Model A \| Model B \| Model C \| Model D \| \| --- \| --- \| --- \| --- \| --- \| --- \| \| Model B \| NRI \| 0.308 (95% CI 0.177 to 0.446) \| — \| — \| — \| \|  \| IDI \| 0.038 (95% CI 0.027 to 0.050) \| — \| — \| — \| \| Model C \| NRI \| 0.236 (95% CI 0.097 to 0.380) \| -0.158 (95% CI -0.287 to -0.017) \| — \| — \| \|  \| IDI \| 0.013 (95% CI 0.004 to 0.021) \| -0.025 (95% CI -0.038 to -0.012) \| — \| — \| \| Model D \| NRI \| 0.374 (95% CI 0.225 to 0.512) \| 0.236 (95% CI 0.097 to 0.380) \| 0.308 (95% CI 0.177 to 0.446) \| — \| \|  \| IDI \| 0.047 (95% CI 0.033 to 0.062) \| 0.009 (95% CI 0.001 to 0.016) \| 0.034 (95% CI 0.024 to 0.045) \| — \| \| Model E \| NRI \| 0.399 (95% CI 0.262 to 0.536) \| 0.227 (95% CI 0.084 to 0.367) \| 0.321 (95% CI 0.182 to 0.466) \| -0.072 (95% CI -0.199 to 0.071) \| \|  \| IDI \| 0.046 (95% CI 0.032 to 0.062) \| 0.008 (95% CI 0.000 to 0.016) \| 0.033 (95% CI 0.023 to 0.044) \| -0.001 (95% CI -0.003 to 0.002) \| \| Model A: Included diabetes, hypertension, low-density lipoprotein, left ventricular ejection fraction, body mass index, B-type natriuretic peptide, troponin T, and fibrinogen. Model B: Model A + Lp(a) Model C: Model A + HCY Model D: Model A + Lp(a) + HCY Model E: Model A + Lp(a) + HCY + Lp(a)*HCY C-Index: Model A, 0.627; Model B, 0.640; Model C, 0.654; Model D, 0.658; Model E, 0.660. Footnotes: NRI, net reclassification improvement; IDI, integrated discrimination improvement; CI, confidence interval. \| \| \| \| \| \| |
| --- | --- | --- | --- | --- | --- | --- | --- | --- | --- | --- | --- | --- | --- | --- | --- | --- | --- | --- | --- | --- | --- | --- | --- | --- | --- | --- | --- | --- | --- | --- | --- | --- | --- | --- | --- | --- | --- | --- | --- | --- | --- | --- | --- | --- | --- | --- | --- | --- | --- | --- | --- | --- | --- | --- | --- | --- | --- | --- | --- | --- |

**Supplementary Table 4. Component-specific MACE analysis.**

|  | **Model 1** | | **Model 2** | | **Model 3** | |
| --- | --- | --- | --- | --- | --- | --- |
|  | **HR (95% CI)** | **P value** | **HR (95% CI)** | **P value** | **HR (95% CI)** | **P value** |
| **Cardiac death (n=23)** |  |  |  |  |  |  |
| Low Lp(a), Low HCY | Reference | | Reference | | Reference | |
| Low Lp(a), High HCY | 4.108 (1.193-14.146) | 0.025* | 4.050 (1.172-13.991) | 0.027* | 4.136 (1.159-14.756) | 0.029* |
| High Lp(a), Low HCY | 7.474 (2.046-27.298) | 0.002** | 7.066 (1.924-25.953) | 0.003** | 6.709 (1.739-25.890) | 0.006** |
| High Lp(a), High HCY | 12.548 (3.529-44.613) | ＜0.001** | 12.360 (3.457-44.196) | ＜0.001** | 11.536 (2.970-44.813) | ＜0.001** |
| **Unplanned coronary revascularization (n=83)** |  |  |  |  |  |  |
| Low Lp(a), Low HCY | Reference | | Reference | | Reference | |
| Low Lp(a), High HCY | 1.138 (0.632-2.049) | 0.667 | 1.169 (0.648-2.111) | 0.603 | 0.990 (0.539-1.817) | 0.974 |
| High Lp(a), Low HCY | 2.334 (1.268-4.296) | 0.006** | 2.320 (1.254-4.291) | 0.007** | 2.202 (1.164-4.166) | 0.015* |
| High Lp(a), High HCY | 3.789 (2.085-6.884) | ＜0.001** | 3.937 (2.156-7.187) | ＜0.001** | 3.769 (2.024-7.018) | ＜0.001** |
| **Heart failure readmission (n=63)** |  |  |  |  |  |  |
| Low Lp(a), Low HCY | Reference | | Reference | | Reference | |
| Low Lp(a), High HCY | 0.658 (0.228-1.902) | 0.440 | 0.648 (0.224-1.877) | 0.424 | 0.634 (0.217-1.851) | 0.404 |
| High Lp(a), Low HCY | 1.487 (0.829-2.665) | 0.183 | 1.525 (0.848-2.741) | 0.159 | 1.357 (0.741-2.484) | 0.323 |
| High Lp(a), High HCY | 2.923 (1.425-5.994) | 0.003** | 2.946 (1.428-6.078) | 0.003** | 2.498 (1.165-5.358) | 0.019 |
| **Nonfatal recurrent MI (n=30)** |  |  |  |  |  |  |
| Low Lp(a), Low HCY | Reference | | Reference | | Reference | |
| Low Lp(a), High HCY | 2.032 (0.817-5.052) | 0.127 | 2.189 (0.875-5.476) | 0.094 | 1.954 (0.757-5.043) | 0.166 |
| High Lp(a), Low HCY | 2.120 (0.645-6.966) | 0.216 | 2.301 (0.689-7.684) | 0.176 | 2.545 (0.754-8.593) | 0.132 |
| High Lp(a), High HCY | 6.248 (2.371-16.462) | ＜0.001** | 6.834 (2.553-18.296) | ＜0.001** | 7.874 (2.863-21.656) | ＜0.001** |
| **Nonfatal stroke (n=25)** |  |  |  |  |  |  |
| Low Lp(a), Low HCY | Reference | | Reference | | Reference | |
| Low Lp(a), High HCY | 0.742 (0.265-2.081) | 0.571 | 0.749 (0.267-2.106) | 0.584 | 0.570 (0.192-1.695) | 0.312 |
| High Lp(a), Low HCY | 0.805 (0.180-3.597) | 0.776 | 0.783 (0.174-3.524) | 0.750 | 0.476 (0.098-2.312) | 0.357 |
| High Lp(a), High HCY | 2.275 (0.746-6.942) | 0.149 | 2.463 (0.803-7.553) | 0.115 | 1.782 (0.535-5.937) | 0.347 |
| Model 1: Adjusted for sex, age; Model 2: Adjusted for Model 1 + body mass index, smoking, drinking, family history of coronary artery disease, killip classification, type of myocardial infarction; Model 3: Adjusted for Model 2 + left ventricular ejection fraction, C-reactive protein, low-density lipoprotein, creatine kinase, troponin T, B-type natriuretic peptide, D-dimer, fibrinogen, multiple vessel disease, percutaneous coronary intervention status. Foototes: *P<0.05, **P<0.01.Low Lp(a) ≤50 mg/dL; high Lp(a) >50 mg/dL; low HCY ≤15 μmol/L; high HCY >15 μmol/L. Lp(a), lipoprotein(a); HCY, homocysteine; HR, hazard ratio; CI, confidence interval. | | | | | | |

**Supplementary Figure 1.** MSRS plot for Lp(a) optimal cutoff.


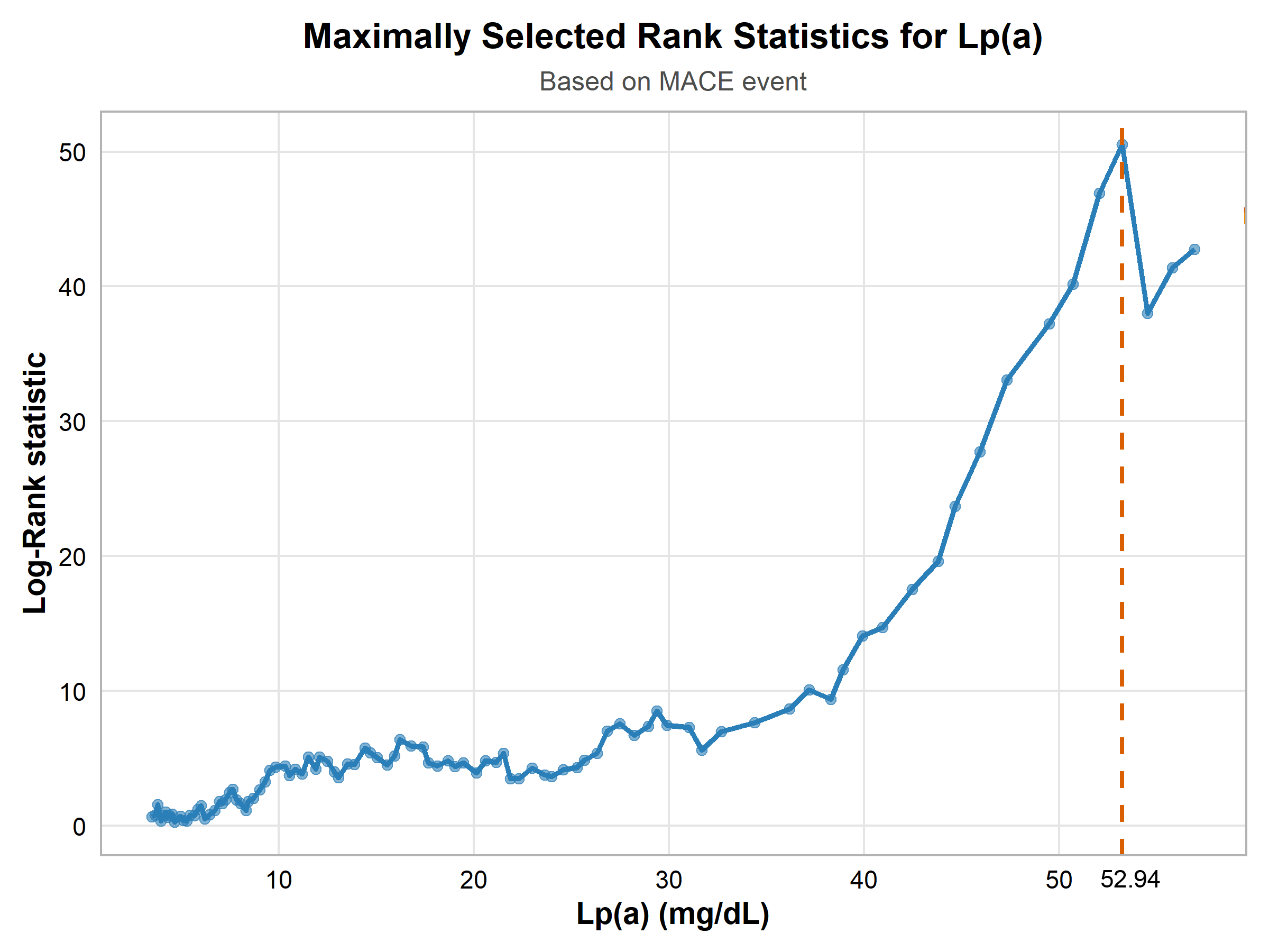


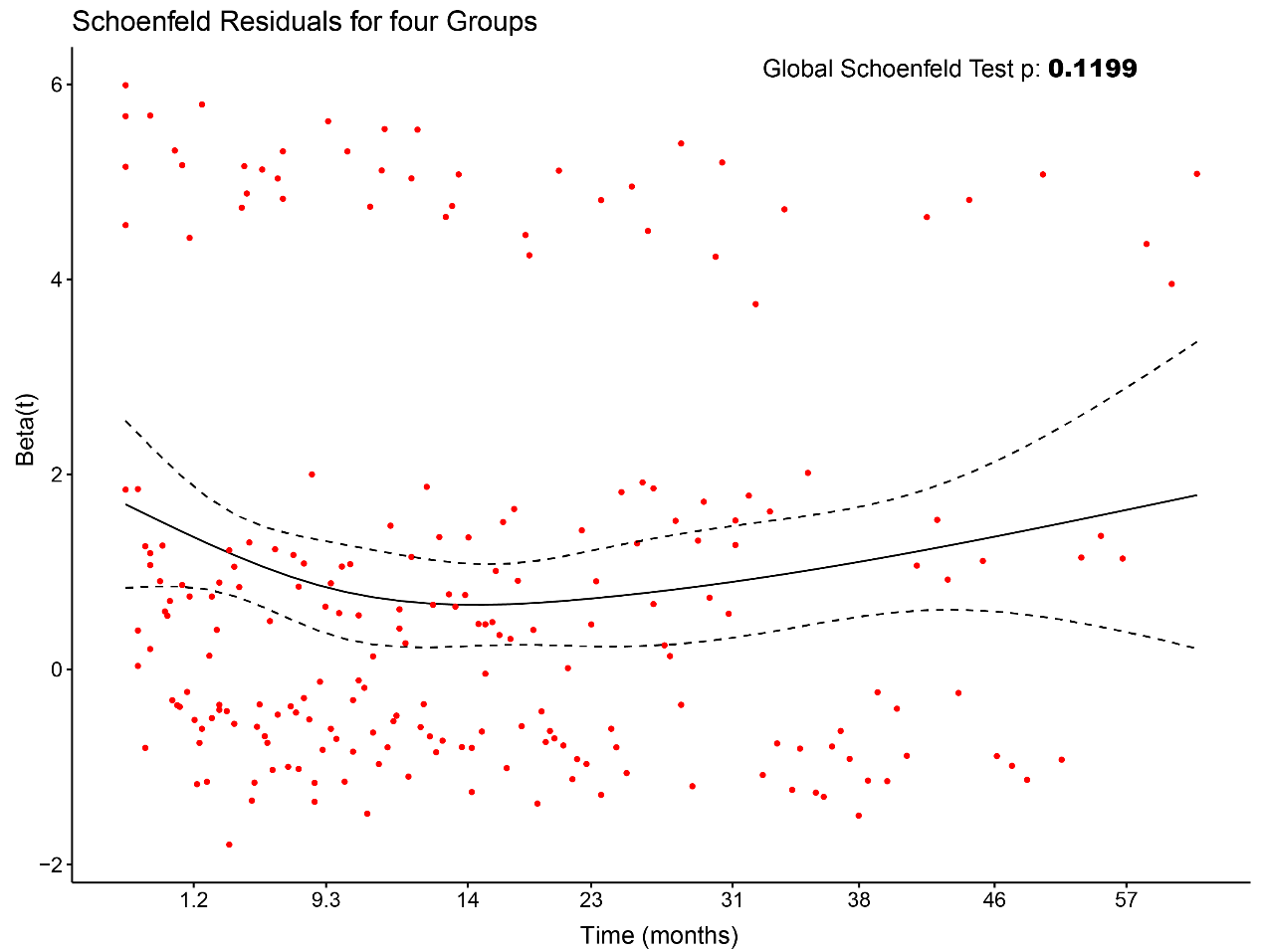
**Supplementary Figure 2.** Schoenfeld residuals for the proportional hazards assumption of the four Lp(a) & HCY groups.
